# Overexpression of Nrf2 Protects against Microcystin-Induced Hepatotoxicity in Mice

**DOI:** 10.1371/journal.pone.0093013

**Published:** 2014-03-25

**Authors:** Yuan-Fu Lu, Jie Liu, Kai Connie Wu, Qiang Qu, Fang Fan, Curtis D. Klaassen

**Affiliations:** 1 University of Kansas Medical Center, Kansas City, Kansas, United States of America; 2 Key Lab for Basic Pharmacology of Ministry of Education, Zunyi Medical College, Zunyi, China; 3 Cytopathology, University of Kansas Medical Center, Kansas City, Kansas, United States of America; Nihon University School of Medicine, Japan

## Abstract

Oxidative stress and glutathione (GSH) depletion are implicated in mycocystin hepatotoxicity. To investigate the role of nuclear factor erythroid 2-related factor 2 (Nrf2) in microcystin-induced liver injury, Nrf2-null, wild-type, and Keap1-hepatocyte knockout (Keap1-HKO) mice were treated with microcystin (50 μg/kg, i.p.). Blood and liver samples were collected 8 h thereafter. Microcystin increased serum alanine aminotransferase and aspartate aminotransferase activities, and caused extensive inflammation and necrosis in Nrf2-null and wild-type mice, but not in Keap1-HKO mice. Oxidative stress and inflammation are implicated in microcystin-induced hepatotoxicity, as evidenced by increased lipid peroxidation and increased expression of pro-inflammatory genes, such as neutrophil-specific chemokines mKC and MIP-2, and pro-inflammatory cytokines IL-1β and IL-6. The increased expression of these pro-inflammatory genes was attenuated in Keap1-HKO mice. Nrf2 and Nqo1 mRNA and protein were higher in Keap1-HKO mice at constitutive levels and after microcystin. To further investigate the mechanism of the protection, hepatic GSH and the mRNA of GSH-related enzymes were determined. Microcystin markedly depleted liver GSH by 60–70% in Nrf2 and WT mice but only 35% in Keap1-HKO mice. The mRNAs of GSH conjugation and peroxide reduction enzymes, such as Gstα1, Gstα4, Gstμ, and Gpx2 were higher in livers of Keap1-HKO mice, together with higher expression of the rate-limiting enzyme for GSH synthesis (Gclc). Organic anion transport polypeptides were increased by microcystin with the most increase in Keap1-HKO mice. In conclusion, this study demonstrates that higher basal levels of Nrf2 and GSH-related genes in Keap1-HKO mice prevented microcystin-induced oxidative stress and liver injury.

## Introduction

Contamination of natural water by cyanobacterial bloom is a worldwide problem, causing public health hazards to humans and livestock [1], [2]. Microcystins, a family of cyclic heptapeptides, are the most common and potent toxins associated with cyanobacteria [3]. Microcystin toxicity has been implicated in liver necrosis, hepatic failure, and liver cancer [4]. The first phase of damage is initiated by the binding of microcystins to sulfhydryls of glutathione (GSH) and proteins, resulting in the generation of reactive oxygen species (ROS) and protein inactivation [5]. Microcystin-induced ROS in turn produce lipid peroxidation and DNA damage [5].

Sulforaphane is an activator of Nrf2 [6] and has been shown to protect against microcystin-induced toxicity to liver cells [7] and livers of mice [6], [7], probably mediated through the activation of Nrf2. Microcystins are known to be detoxified by glutathione to form glutathione conjugates [8]–[10], catalyzed by GSH *S*-transferases in laboratory animals [10] and in humans [11]. Thus, any means that increases cellular GSH and GSH conjugation should be beneficial to protect against microcystin toxicity.

Nuclear factor erythroid 2-related factor 2 (Nrf2) is a major defense mechanism against oxidative and/or electrophilic stress in the liver [12]. Nrf2 is bound by kelch-like ECH associating protein 1 (Keap1) in the cytosol [13]. Genetically knock-down of Keap1 (Keap1-Kd) results in mice with Nrf2 activation throughout the body [14]. By crossing Keap1-Kd mice and AlbCre+ mice, which express Cre only in hepatocytes, Keap1-hepatocyte knockout (Keap1-HKO) mice with maximum Nrf2 activation in liver were generated [15]–[17]. Genetic Nrf2 activation in turn enhances the expression of cytoprotective genes/proteins that catalyze GSH synthesis (Gclc), GSH peroxidase (Gpx), GSH conjugation reactions (Gst), oxidized GSH and other SH-protein repair genes; Genetic Nrf2 activation also increases the expression of NAD(P)H:quinone oxidoreductase 1 (Nqo1) and increases the mRNA expression of hepatic Mrp transporters [15], [17]. We have used this genetic Nrf2 “gene-dose” model to examine the effect of Nrf2 activation on the hepatotoxicity of 13 chemicals. Nrf2 has shown to protect against the hepatotoxicity produced by 10 of the hepatotoxicants including microcystin [18], but the underlining mechanisms remain to be elucidated.

Thus, the purpose of the present study was to determine the role of constitutive activation of Nrf2 in the protection against microcystin acute toxicity using the Nrf2 “gene dose–response” model, focusing on hepatic GSH, GSH conjugation, as well as oxidized GSH and SH-protein reducing enzymes.

## Materials and Methods

### Reagents

Microcystin (Microcystin-LA) was purchased from Sigma-Aldrich (St. Louis, MO). All other chemicals were reagent grade and commercially available.

### Animals

Nrf2-null mice were obtained from Dr. Jefferson Chan (University of California, Irvine, CA) [19], the genetic background C57BL/6 mice were purchased from Charles River Laboratories, Inc. (Wilmington, MA), and Keap1-HKO mice [15], [17] were backcrossed at least eight generations and shown to be congenic by Jackson Laboratories. Mice were housed in a temperature-, light-, and humidity-controlled facilities and had free access to standard rodent chow and water *ad libitum*. Animal care was provided in accordance with the US Public Health Policy on the Care and Use of Animals, and the study protocol was approved by the Institutional Animal Care and Use Committee of the University of Kansas Medical Center.

### Experimental Design

Nrf2-null mice, wild-type mice, and Keap1-HKO mice were treated with either microcystin-LA (50 μg/kg, i.p.) or saline (10 ml/kg, i.p.). Blood and livers were collected 8 hrs thereafter. Portions of livers were fixed in 10% neutral formalin for histological analysis, and others were frozen in liquid nitrogen and stored at −80°C. The dose and time selection were based on our previous publication [20] and pilot experiments.

### Hepatotoxicity Evaluation

Blood was placed on ice for 60 min and centrifuged (8000 *g*, 10 min) to prepare serum. Serum samples were analyzed by standard enzymatic assays using commercial kits for alanine aminotransferase (Infinity ALT) and aspartate aminotransferase (Infinity AST, Thermo Scientific, Middletown, VA) as indicators of hepatotoxicity.

### Histopathology

A portion of each liver was fixed in 10% neutral formalin, processed by standard histological techniques, and stained with hematoxylin and eosin (H&E). and evaluated for hepatocellular necrosis with a Zeiss microscope.

### Lipid Peroxidation

Lipid peroxidation in livers was determined by quantifying thiobarbituric acid reactive substances (TBARS), using malondialdehyde (MDA) as the standard (Thermo Fisher Scientific Inc., Fair Lawn, NJ).

### Quantification of GSH Concentrations

The concentrations of reduced glutathione (GSH) were estimated by total thiol levels in livers of mice using a 5,5′-dithiobis-2-nitrobenzoic acid assay and the thionitrobenzene formed was quantified spectrophotometrically using a GSH assay kit (CS0260) from Sigma (St Louis, MO).

### Total RNA Isolation and RT-PCR

Total RNA was isolated using RNAzol B reagent (Tel-Test, Inc., Friendswood, TX) and quantified spectrophotometrically at 260 nm. The quality of RNA samples were evaluated by 260/280 ratios (>1.8). Total RNA was reverse-transcribed into cDNA by a High Capacity RT Kit (Applied Biosystems, Foster City, CA), and amplified with Power SYBR Green PCR Master Mix in a 7900HT PCR System (Applied Biosystems, Foster City, CA). Oligonucleotide primers were designed with Primer3 software, and are listed in Table S1. The expression of genes was calculated by the 2^−ΔΔCt^ method, normalized to the house-keeping gene G3PDH, and expressed as relative expression of%G3PDH.

### Western Blot Analysis

Liver protein was extracted with a T-PER tissue protein extraction kit (Thermo Scientific, Rockford, IL) with freshly prepared proteinase inhibitors (Sigma, St. Louis, MO). Protein concentrations were determined using a BCA protein assay according to the manufacturer’s instructions (Thermo Scientific, Rockford, IL). Approximately 40 μg of cytosolic protein was used for immunoblotting proteins of interest. The primary antibodies used in this study include Gclc (sc-27688) from Santa Cruz Biotechnology (Santa Cruz, CA), whereas Nqo1 (Ab2346) and β-actin (Ab8227) were from Abcam (Cambridge, MA). Secondary antibodies were purchased from Sigma-Aldrich (St. Louis, MO). Protein-antibody complexes were detected using an enhanced chemiluminescent kit (Thermo Scientific, Rockford, IL) and exposed to HyBlot CL autoradiography film (Denville Scientific Inc., Metuchen, NJ).

### Statistical Analysis

Data are expressed as mean ± SEM and analyzed using a one-way ANOVA followed by Duncan’s multiple range test utilizing SPSS 13 Software (SAS, NC). The significant level was set at p ≤ 0.05.

## Results

### Nrf2 Protects Against Hepatotoxicity of Microcystin

**Figure 1:**
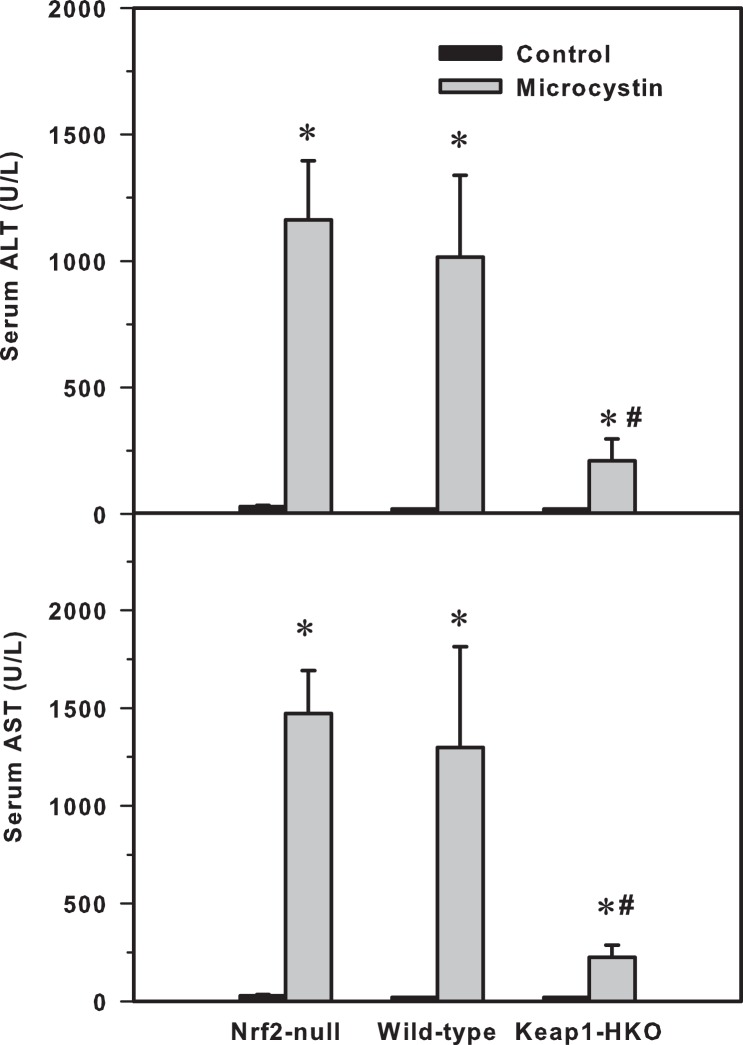
(Top) presents serum alanine aminotransferase (ALT), (Bottom) serum aspartate aminotransferase (AST) in Nrf2-null, wild-type and Keap1-HKO mice administered saline (10 ml/kg, i.p.) or microcystin (50 μg/kg, i.p.). Values are expressed as mean ± S.E.M. (n = 7–10). *Significantly different from the basal level of the same genotype (p ≤ 0.05); #Significantly different from Nrf2-null mice treated with microcystin (p ≤ 0.05).

Microcystin-induced acute hepatotoxicity was indicated by increased serum enzyme activities of alanine aminotranferase (ALT) and aspartate aminotrasferase (AST). In saline-treated mice, serum ALT and AST activities in Nrf2-null, wild-type, and Keap1-HKO mice were low, without differences between genotypes. Microcystin increased serum ALT activities 45-fold in Nrf2-null mice, 42-fold in wild-type mice, but only 5.4-fold in in Keap1-HKO mice (Figure 1, top). Serum AST activities of Nrf2-null, wild-type, and Keap1-HKO mice showed a similar pattern, that is, microcystin increased serum AST activities 47-fold in Nrf2-null mice, 45-fold in wild-type mice, but only 7.5-fold in Keap1-HKO mice (Figure 1, bottom).

#### Liver histopathology

**Figure 2:**
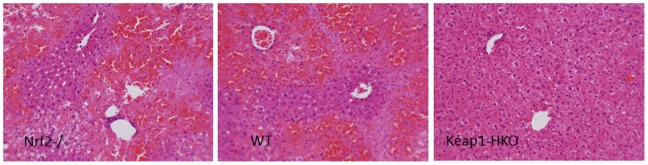
Histological analysis of livers from Nrf2-null, wild-type, and Keap1-HKO mice treated with microcystin (50 μg/kg, i.p.). H & E staining with magnification (200×).

There were no observable abnormalities in the livers of control Nrf2-null, wild-type, and Keap1-HKO mice (data not shown). However, microcystin produced severe hemorrhage, inflammation and extensive necrosis in Nrf2-null mice as we observed earlier [20]. Similar lesions were also evident in wild-type mice, but to a lesser extent. In comparison, only mild hepatocyte swelling was evident in Keap1-HKO mice (Figure 2), and no apparent necrosis was observed.

### Nrf2 Prevented GSH Depletion and Reduced Microcystin-induced Lipid Peroxidation

Microcystin has been reported to produce a decrease of GSH in the liver as early as 2 hrs [10] and ROS generation within 12 hrs after exposure [5]. Thus, both hepatic GSH and lipid peroxidation levels were quantified.

#### GSH

**Figure 3:**
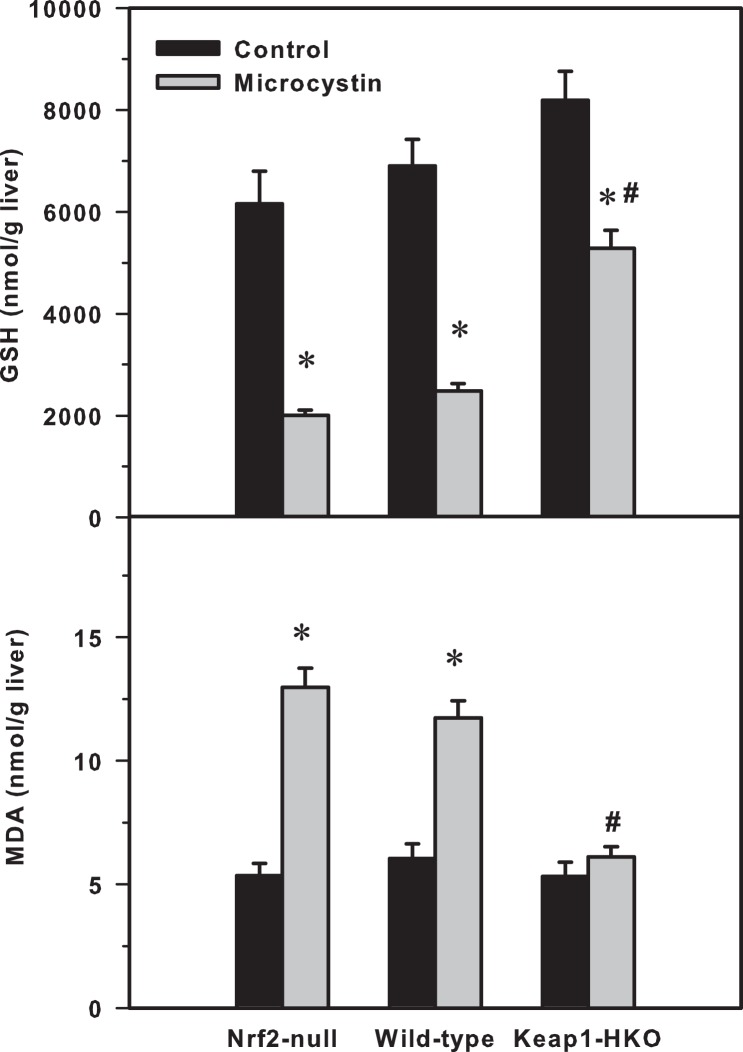
(Left) Hepatic glutathione concentrations (GSH), (Right) hepatic malondialdehyde (MDA) levels in Nrf2-null, wild-type and Keap1-HKO mice administered saline (10 ml/kg, i.p.) or microcystin (50 μg/kg, i.p.). Values are expressed as mean ± S.E.M. (n = 5–8). *Significantly different from the basal level of the same genotype (p ≤ 0.05); #Significantly different from Nrf2-null mice treated with microcystin (p ≤ 0.05).

The basal GSH concentrations in liver tended to be higher in mice with graded Nrf2 activation. After microcystin injection, the hepatic GSH concentrations decreased by 70% in Nrf2-null mice, 64% in wild-type mice, but only 35% in Keap1-HKO mice, when compared to their corresponding controls (Figure 3, left panel).

#### TBARS

Figure 3, right panel, shows the amount of lipid peroxidation, as determined by thiobarbiturate reactive substances (TBARS), using malondialdehyde (MDA) as the standard, in the livers of the various groups of mice. Microcystin increased hepatic MDA levels 117% in Nrf2-null mice, 82% in WT mice, and 24% in the Keap1-HKO mice.

### Gene Expression Analysis

#### Nrf2-target genes Nqo1 and Gclc

**Figure 4:**
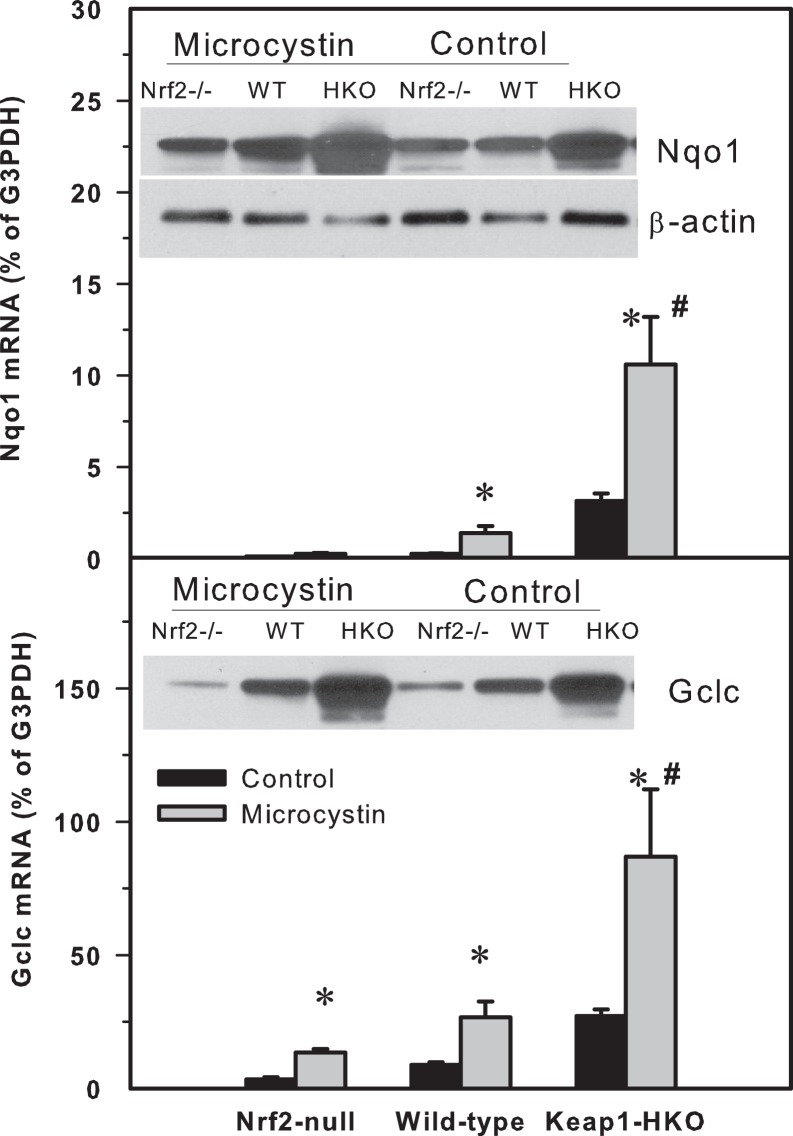
(Top) The mRNA levels of NAD(P)H quinone oxidoreductase 1 (Nqo1) and glutamate-cysteine ligase, catalytic subunit (Gclc) in Nrf2-null, wild-type and Keap1-HKO mice administered saline (10 ml/kg, i.p.) or microcystin (50 μg/kg, i.p.). Livers were removed 8-PCR. Values are expressed as mean ± S.E.M. (n = 8). *Significantly different from the basal level of the same genotype (p ≤ 0.05); #Significantly different from Nrf2-null mice treated with microcystin (p ≤ 0.05). Representative Western-blot analysis of Nqo1 and Gclc proteins inserted into mRNA expression graphs. Nqo1 and Gclc were higher in Keap1-HKO mice at both basal levels and after microcystin.

To determine the mechanism of how Nrf2 protects against microcystin-induced toxicity, expression of the Nrf2-target genes NAD(P)H quinone oxidoreductase 1 (Nqo1) and glutamate-cysteine ligase, catalytic subunit (Gclc) were quantified in livers of Nrf2-null, wild-type, and Keap1-HKO mice (Figure 4). The basal levels (% of G3PDH) of Nqo1 were 0.137, 0.293 and 3.173 for Nrf2-null, WT, and Keap1-HKO mice, respectively. Eight hours after microcystin administration, mRNA of Nqo1 increased to 0.281, 1.402 and 11.63 for Nrf2-null, WT, and Keap1-HKO mice, respectively. The basal levels of Gclc were 3.843, 8.825 and 27.24 for Nrf2-null, WT, and Keap1-HKO mice, respectively; Eight hours after microcystin administration, mRNA of Gclc increased to 13.63, 26.08 and 86.89 for the Nrf2-null, WT, and Keap1-HKO mice, respectively, indicating that Nrf2-targeted genes Nqo1 and Gclc were increased after microcystin in a “gene-dose” manner.

The representative western-blot analysis of Nqo1 and Gclc protein expression was inserted into Figure 4. Consistent with gene expression analysis, the expression of Nqo1 and Gclc proteins were highest in Keap1-HKO mouse livers, both before and after microcystin intoxication.

#### Chemokine genes

**Figure 5:**
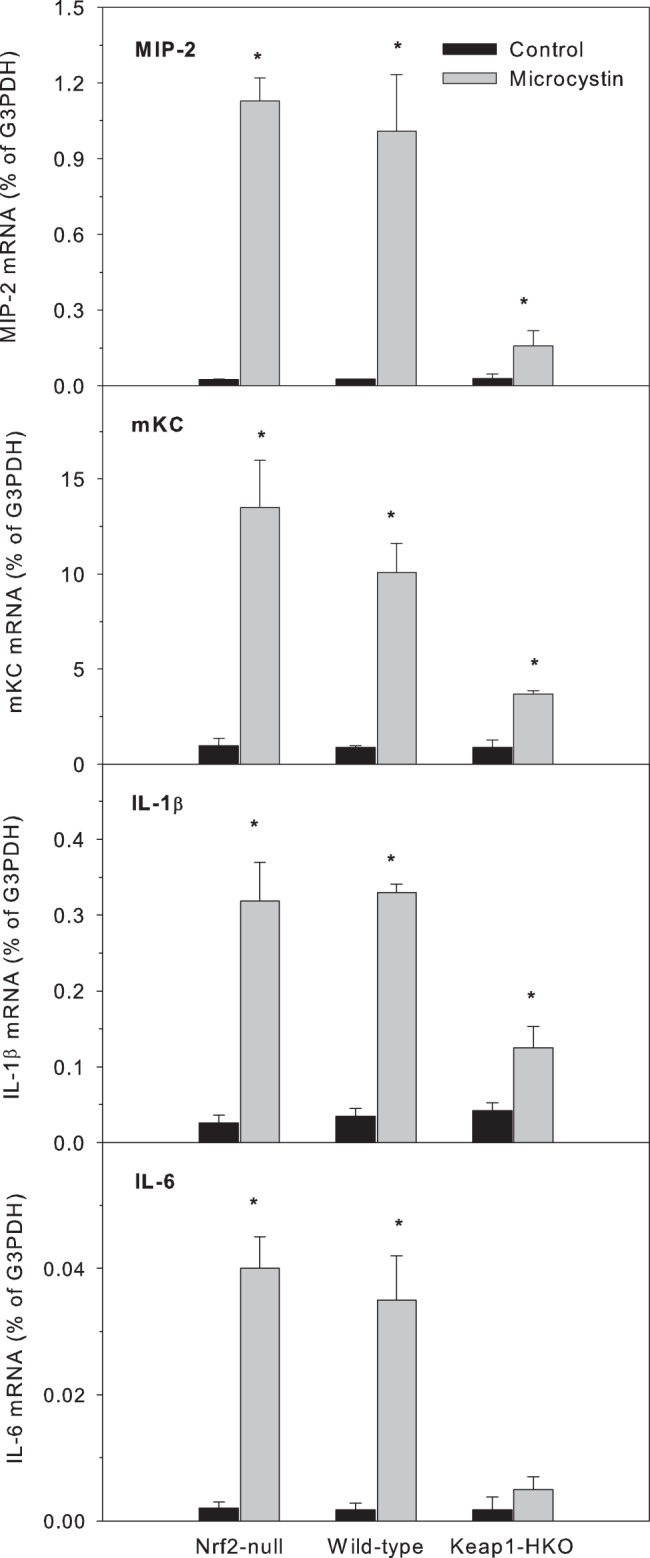
The mRNA levels of neutrophil-specific chemokine macrophage inflammatory protein 2 (MIP-2), mouse keratinocyte-derived chemokine (mKC), inflammation genes interleukin-1β (IL-1β) and IL-6 in Nrf2-null, wild-type and Keap1-HKO mice administered saline (10 ml/kg, i.p.) or microcystin (50 μg/kg, i.p.). Livers were removed 8-PCR. Values are expressed as mean ± S.E.M. (n = 8). *Significantly different from the basal level of the same genotype (p ≤ 0.05); #Significantly different from Nrf2-null mice treated with microcystin (p ≤ 0.05).

mRNA levels of neutrophil-specific chemokine macrophage inflammatory protein 2 (MIP-2) and mouse keratinocyte-derived chemokine (mKC) are shown in Figure 5, left panel. There were no differences in basal expression of MIP-2 (around 0.03% of G3PDH) among the three genotypes. Eight hours after microcystin administration, the mRNA of MIP-2 increased 45-fold, 37-fold, and 5.5-fold for the Nrf2-null, WT, and Keap1-HKO mice, respectively. There were also no differences in basal expression of mKC (around 1% of G3PDH) among the three genotypes. Eight hours after microcystin administration, the mRNA of mKC increased 14-fold, 12-fold, and 4-fold for Nrf2-null, WT, and Keap1-HKO mice, respectively, indicating that Nrf2-overexpression attenuated the microcystin-induced inflammatory response in a “gene-dose” manner.

#### Pro-inflammatory cytokine genes

The mRNA levels of pro-inflammatory genes interleukin-1β (IL-1β) and IL-6 are shown in Figure 5, right panel. There were no differences among the three genotypes in the basal expression of IL-1β (0.04% of G3PDH). Microcystin markedly increased the mRNA of IL-1β by 12-fold in Nrf2-null mice, 10-fold in WT mice, and 3-fold in Keap1-HKO mice. There were no differences among the three genotypes in the basal expression of IL-6 (0.002% of G3PDH). Microcystin increased the mRNA of IL-6 20-fold in Nrf2-null and WT mice, and only 3-fold in Keap1-HKO mice. Similar results were evident for tumor necrosis factor alpha (TNFα) (data not shown). These data indicate that constitutive Nrf2 activation attenuated microcystin-induced hepatic inflammation.

**Figure 6:**
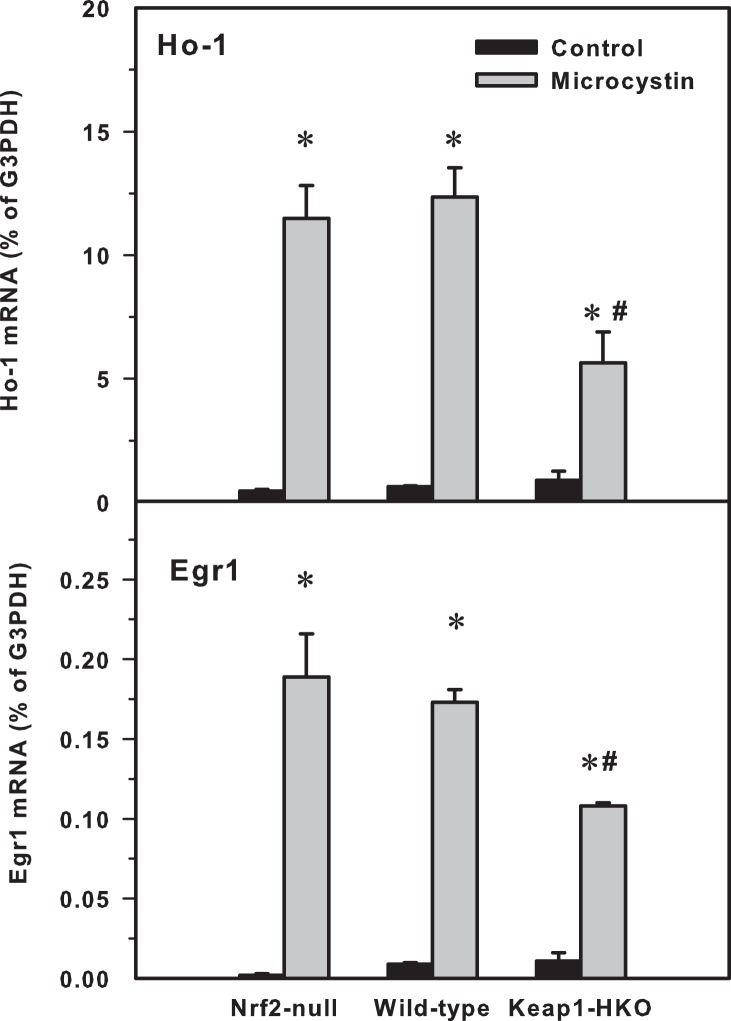
The mRNA levels of glutathione conjugation enzymes Gstα1, Gstα4, Gtsμ and Gpx2 in Nrf2-null, wild-type and Keap1-HKO mice administered saline (10 ml/kg, i.p.) or microcystin (50 μg/kg, i.p.). Livers were removed 8-PCR. Values are expressed as mean ± S.E.M. (n = 8). *Significantly different from the basal level of the same genotype (p ≤ 0.05); #Significantly different from Nrf2-null mice treated with microcystin (p ≤ 0.05).

In accordance with the inflammatory response, the expression of the acute phase proteins Ho-1 (Heme oxygenase −1) and Egr1 (Early growth response protein 1) were also increased more in Nrf2-null and wild-type mice than in Keap1-HKO mice (Figure 6).

#### Genes involved in GSH conjugation and peroxide reduction

**Figure 7:**
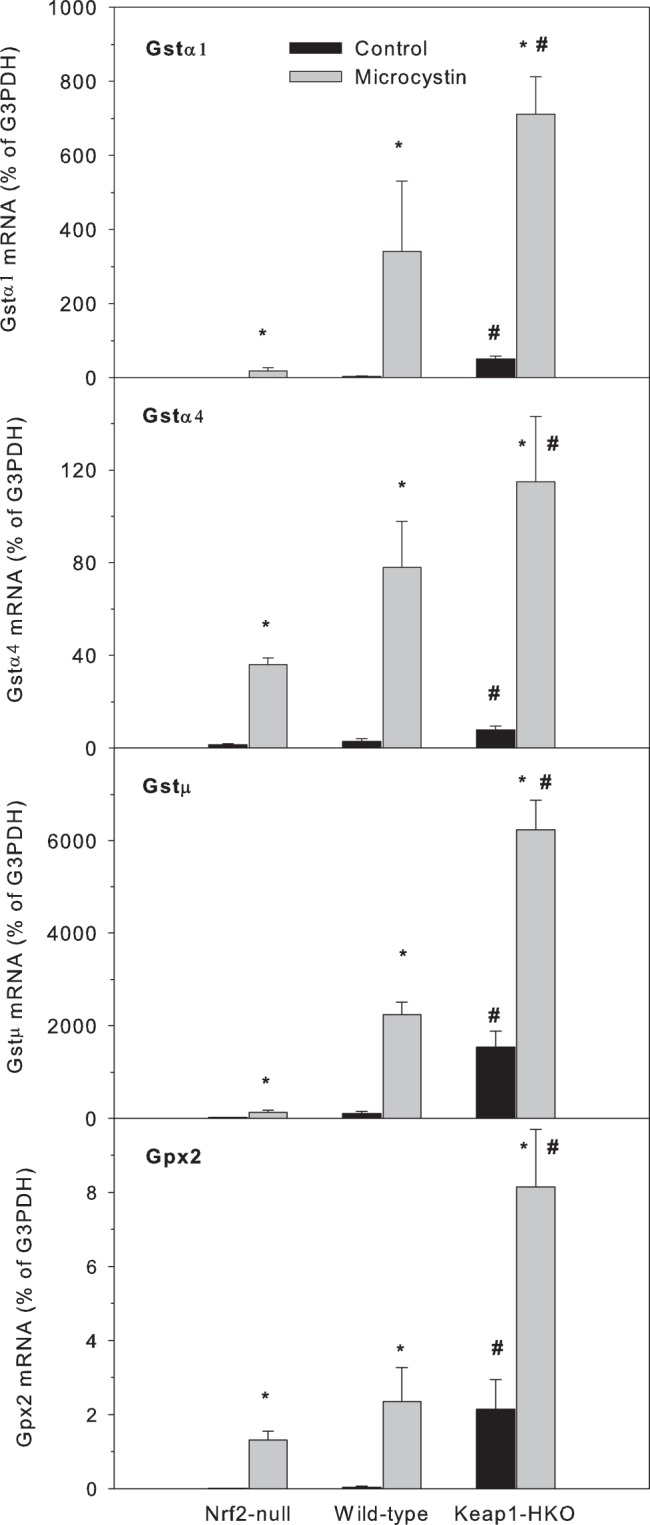
mRNA levels of heme oxygenase-1 (Ho-1, top) and early growth response protein-1 (Egr1) in Nrf2-null, wild-type and Keap1-HKO mice administered saline (10 ml/kg, i.p.) or microcystin (50 μg/kg, i.p.). Values are expressed as mean ± S.E.M. (n = 8). *significantly different from the basal level of the same genotype (p ≤ 0.05); #significantly different from Nrf2-null mice treated with microcystin (p ≤ 0.05).

The mRNAs of enzymes involved in GSH conjugation and peroxide reduction before and after microcystin administration are shown in Figure 7 (Gstα1, Gstα4 and Gtsμ for GSH conjugation and Gpx2 for peroxide reduction). In general, the mRNA for these GSH transferase enzymes in the basal condition were expressed at low levels, but markedly induced by microcystin. Consistent with our recent publication (Wu et al., 2012b), much higher basal expression was seen in Keap1-HKO mice compared to Nrf2-null mice for Gstα1(113-fold), Gstα4 (5.3-fold), Gstμ (77-fold) and Gpx2 (108-fold). Microcystin further increased the expression of these genes, but larger increases were evident in Keap1-HKO mice compared to Nrf2-null mice for Gstα1(39-fold), Gstα4 (33-fold), Gtsμ (48-fold) and Gpx2 (6.2-fold). The expression of these genes in wild-type mice was between that in Nrf2-null and Keap1-HKO mice. Thus, the induction of Gsts is apparently a major defense mechanism in response to microcystin, and Keap1-HKO mice showed a much higher increase in Gst and Gpx2 mRNAs, together with higher hepatic GSH concentrations (Figure 3).

#### Genes involved in microcystin uptake

**Figure 8:**
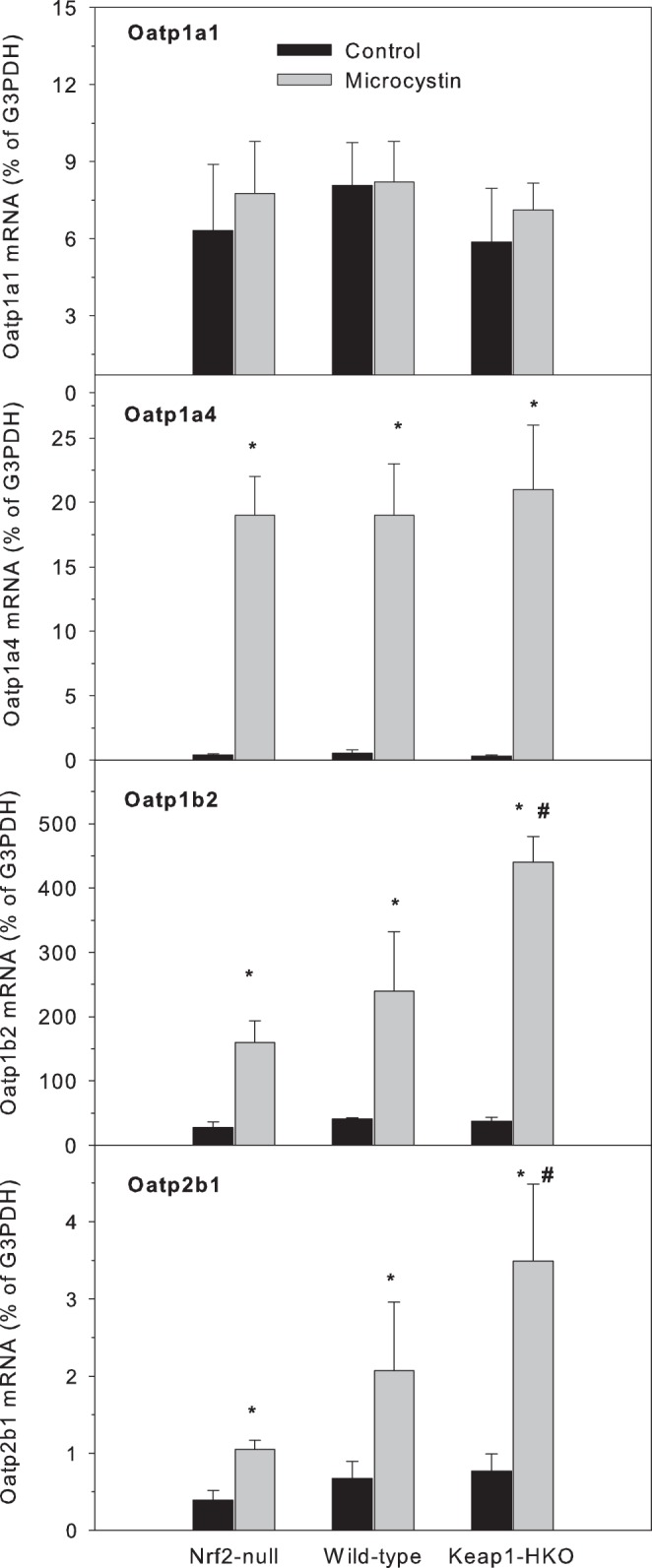
Expression of hepatic organic anion transporting peptide Oatp1a1, Oatp1a4, Oatp1b2, and Oatp2b1 in Nrf2-null, wild-type and Keap1-HKO mice administered saline (10 ml/kg, i.p.) or microcystin (50 μg/kg, i.p.). Livers were removed 8-PCR. Values are expressed as mean ± S.E.M. (n = 8). *Significantly different from the basal level of the same genotype (p ≤ 0.05); #Significantly different from Nrf2-null mice treated with microcystin (p ≤ 0.05).

Oatp transporters have been reported to play an important role in microcystin transport into cells [20]–[22]. In the present study, there was no difference in Oatp1a1 mRNA expression before or after microcystin among the three genotypes. Oatp1a4 was induced more than 50-fold by microcystin in all three genotypes of mice, but there was no difference between Nrf2-null and Keap1-HKO mice (Figure 8). In comparison, although the basal expression of Oatp1b2 (homologue to human OATP1B1 and OATP1B3) is not different among the three genotypes of mice, microcystin induced Oatp1b2 mRNA 5.9-fold in Nrf2-null mice, and increased 11.9-fold in Keap1-HKO mice. Expression of Oatp2b1 showed a similar pattern: microcystin increased Oatp2b1 approximately 3-fold in Nrf2-null mice and wild-type mice, but 4.5-fold in Keap1-HKO mice.

## Discussion

In the present study, microcystin induced acute hepatotoxicity is evident by increased serum activities of ALT and AST, as well as widespread liver hemorrhage and hepatocellular necrosis. Although there was no statistical difference in microcystin sensitivity between Nrf2-null mice and wild-type mice, overexpression of Nrf2 did protect against microcystin toxicity. This is probably due to extensive liver damage produced by this dose of microcystin that overwhelmed the defense mechanism in wild-type mice, as this phenomenon also occurred for the hepatotoxicity produced by phalloidin, cadmium, bromobenzene and ally alcohol [18].

Microcystin is rapidly taken up by hepatocytes through carrier-mediated transport. Oatp transporters, such as OATP1B1 and OATP1B3 in human cells, have been reported to play an important role in microcystin transport into cells [21], [22], and Oatp1b2-null mice transport less microcystin into the liver and less liver injury is observed [20]. In the present study, there was no significant difference in the basal expression of Oatp1a1, Oatp1a4, Oatp1b2 and Oatp2b1 among the various Nrf2-genotypes. Microcystin induced the expression of Oatp1a4, Oatp1b2 and Oatp2b1, but not Oatp1a1 (Figure 8). Following microcystin administration, higher expression of Oatp1b2 was evident in Keap1-HKO mice, suggesting that the protection of graded Nrf2 activation against microcystin toxicity is not due to diminished uptake of the toxicant into the hepatocytes, as in Oatp1b2-null mice [20].

Microcystin is thought to activate the Keap1–Nrf2 pathway through multiple mechanisms. For example, microcystin increases the Nrf2-target genes Nqo1 and Ho-1 in NIH3T3 cells and HepG2 cells [7], and addition of microcystin to hepatoma HepG2 and Hep3B cells results in microcystin uptake into hepatocytes and the release of Nrf2 into the nucleus [23]. Microcystin causes structural changes in hepatocytes by inhibiting protein phosphorylase 1 and 2A, which produces further cell damage and Nrf2 activation [4]. Thus, microcystin is an activator of Nrf2 in liver, either by microcystin at nontoxic concentration or by microcystin-induced toxicity. Induction of Nrf2 is not a cause of microcystin-induced toxicity, but rather an adaptive mechanism in protecting against microcystin-induced liver injury.

The neutrophil-specific chemokine macrophage inflammatory protein 2 (MIP-2, CXCR2) and mouse keratinocyte-derived chemokine (mKC) are important mediators of inflammation in acute tissue injury [24]. In the present study, microcystin-increased MIP-2 and mKC mRNA in mice, but was much less in Keap1-HKO mice, suggesting that constitutive activation of Nrf2 reduces microcystin-induced liver injury by reducing chemokine activation, resulting in reduced release of inflammatory mediators. Interleukin-1beta (1L-1β), interleukin-6 (IL-6), and tumor necrosis factor alpha (TNFα), are important pro-inflammatory cytokines in acute and chronic liver damage [25], and likely are involved in microcystin-induced liver injury. In the present study, Keap1-HKO mice had much lower expression of these cytokines after administration of microcystin, indicating that Nrf2 reduction of microcystin-induced liver injury could be due, at least in part, to the reduced pro-inflammatory cytokine release in the liver.

Ho-1 and Egr-1 are two cellular protective proteins that respond to toxic stimuli [18], [26]. Induction of these acute phase proteins would be theoretically beneficial to reduce microcystin toxicity. However, over-expression of these proteins can also be envisioned as a potential biomarker of increased tissue damage, and exacerbates microcystin-induced acute hepatotoxicity. In the present study, both Ho-1 and Egr-1 were induced markedly by microcystin (Fig. 6). Although both genes are also considered Nrf2-target genes [17], [18], Ho-1 and Egr1 overexpression in the current study could be considered as biomarkers of microcystin-induced severe liver injury.

ROS play an important role in microcystin-induced acute liver injury. Consistent with the literature, the increased lipid peroxidation 8 hrs after microcystin indicates the involvement of ROS in microcystin hepatotoxicity [5]. In combating against microcystin-induced oxidative stress, the mRNA of GSH synthesis and conjugating enzymes in all three genotype of mice increase, and much more induction was seen in the Keap1-HKO mice. These findings are in agreement with the literature regarding early increases in the mRNAs of GSH synthesis and conjugation enzymes in response to microcystin [9]. However, as the liver damage progresses, the mRNAs of these GSH synthesis and conjugation enzymes might decrease [10]. The present study clearly shows that Keap1-HKO hepatocytes not only have a marked activation of Nrf2, but also a lower level of ROS production following microcystin administration. Apparently, activation of Nrf2 protects against microcystin-induced generation of ROS, similar to that observed for Nrf2 protection against cadmium toxicity [16]. Taken together, GSH concentrations in the liver (Figure 3), the mRNA for the GSH conjugation and peroxide-reduction enzymes (Figure 7), and the mRNA for rate-limiting enzyme for GSH synthesis Gclc (Figure 4) were expressed at higher levels with graded Nrf2 activation in the Nrf2 “gene-dose” model [16], [17], not only at basal levels, but also at much higher levels after microcystin challenge. Thus, enhancement of GSH synthetic, conjugating and peroxide reduction enzymes appear to be important for reducing inflammation and oxidative stress in mice with genetic over-expression of Nrf2.

In conclusion, the present study demonstrates that Nrf2 activation decreases microcystin-induced oxidative stress and liver injury. The protective effects of Nrf2 appear to depend on higher expression of genes involved in antioxidant defense, particularly the enhanced GSH synthetic, conjugation and peroxide reduction enzymes.

## Supporting Information

Table S1 **Oligonucleotide sequences for primers specific for RT-PCR analysis.**
(DOCX)
