# Supplementary material for: Overexpression of Nrf2 Protects against Microcystin-Induced Hepatotoxicity in Mice
Source: PLoS One. 2014 Mar 25;9(3):e93013. doi: 10.1371/journal.pone.0093013 (PMC3965536; doi:10.1371/journal.pone.0093013)
Supplement: Table S1 — Oligonucleotide sequences for primers specific for RT-PCR analysis. (DOCX) [file pone.0093013.s001.docx]

Table S1: Oligonucleotide sequences for primers specific for RT-PCR analysis.
